# Factors associated with readmissions in women participating in screening programs and treated for breast cancer: a retrospective cohort study

**DOI:** 10.1186/s12913-019-4789-3

**Published:** 2019-12-05

**Authors:** Carme Miret, Laia Domingo, Javier Louro, Teresa Barata, Marisa Baré, Joana Ferrer, Maria Carmen Carmona-García, Xavier Castells, Maria Sala

**Affiliations:** 1 Preventive Medicine and Public Health Training Unit PSMar-UPF-ASPB, Parc de Salut Mar, Agència de Salut Pública de Barcelona, Pompeu Fabra University, Barcelona, Spain; 2 Department of Epidemiology and Evaluation, IMIM (Hospital del Mar Medical Research Institute), Passeig Marítim, 25-29, 08003 Barcelona, Spain; 3 Department of Pediatrics, Obstetrics and Gynecology, Preventive Medicine and Public Health, Universitat Autònoma de Barcelona (UAB), 08193 Bellaterra, Barcelona, Spain; 4 Research Network on Health Services in Chronic Diseases (REDISSEC), Av. de Monforte de Lemos, 5, 28029 Madrid, Spain; 5 General Directorate of Health Care Programs, Canary Islands Health Service, C/ Juan XXIII,13, 35005 Las Palmas de Gran Canaria, Spain; 6 Cancer Screening and Clinical Epidemiology, Corporació Sanitària Parc Taulí, 08208 Sabadell, Spain; 7 Department of Radiology, Hospital de Santa Caterina, C/ Dr. Castany, s/n, 17190 Salt, Girona, Spain; 8 Epidemiology Unit and Girona Cancer Registry, Oncology Coordination Plan, Department of Health, Catalan Institute of Oncology, C/ Sol, 15, 17004 Girona, Spain; 9 Girona Biomedical Research Institute (IDIBGI), C/ Dr Castany s/n, 17190 Salt, Girona, Spain; 10 Department of Medical Oncology, Catalan Institute of Oncology, University Hospital Dr Josep Trueta, Av. França, S/N, 17007 Girona, Spain

**Keywords:** Breast cancer, Screening, Readmission, Complication

## Abstract

**Background:**

We aimed to identify the risk factors associated with early, late and long-term readmissions in women diagnosed with breast cancer participating in screening programs.

**Methods:**

We performed a multicenter cohort study of 1055 women aged 50–69 years participating in Spanish screening programs, diagnosed with breast cancer between 2000 and 2009, and followed up to 2014. Readmission was defined as a hospital admission related to the disease and/or treatment complications, and was classified as early (< 30 days), late (30 days-1 year), or long-term readmission (> 1 year). We used logistic regression to estimate the adjusted odds ratios (aOR), and 95% confidence intervals (95% CI) to explore the factors associated with early, late and long-term readmissions, adjusting by women’s and tumor characteristics, detection mode, treatments received, and surgical and medical complications.

**Results:**

Among the women included, early readmission occurred in 76 (7.2%), late readmission in 87 (8.2%), long-term readmission in 71 (6.7%), and no readmission in 821 (77.8%). Surgical complications were associated with an increased risk of early readmissions (aOR = 3.62; 95%CI: 1.27–10.29), and medical complications with late readmissions (aOR = 8.72; 95%CI: 2.83–26.86) and long-term readmissions (aOR = 4.79; 95%CI: 1.41–16.31).

**Conclusion:**

Our results suggest that the presence of surgical or medical complications increases readmission risk, taking into account the detection mode and treatments received. Identifying early complications related to an increased risk of readmission could be useful to adapt the management of patients and reduce further readmissions.

**Trial Registration:**

ClinicalTrials.govIdentifier: NCT03165006. Registration date: May 22, 2017 (Retrospectively registered).

## Background

Breast cancer is the most frequently diagnosed malignant tumor and the main cause of cancer death in women worldwide [1, 2]. However, over the last 20 years, there has been a reduction in breast cancer mortality rates in Western countries, which has mainly been attributed to the widespread use of breast cancer screening practices, advances in adjuvant treatments, and improvements in healthcare quality, especially through the introduction of functional units and multidisciplinary teams in hospitals [3–5]. This mortality reduction implies an increasing number of cancer survivors who face the possibility of experiencing a breast cancer recurrence, another cancer diagnosis, or adverse effects of treatment, which translates into increased use of health services [6].

Women participating in breast cancer screening programs are more likely to be diagnosed at earlier stages [7, 8], showing better survival [7, 9, 10] and requiring less aggressive treatment modalities [11, 12] than women diagnosed symptomatically, and have higher percentages of breast-conserving surgery [11]. Treatment approaches for patients with early-stage and locally advanced breast cancer require a multidisciplinary treatment approach that may include surgery, radiation therapy, and systemic therapy. Whereas the impact of screening and early detection have been widely evaluated in terms of recurrences and mortality [5, 13], less attention has been paid to their impact on readmissions and treatment-related complications.

Most studies on readmissions in women with breast cancer have been conducted in the US context and have focused on short-term readmissions after surgery [14–18], without considering the broader therapeutic approach employed in women with breast cancer. Only a couple of studies have evaluated readmissions due to the complications of adjuvant treatment received by patients with breast cancer [19, 20]. In addition, to our knowledge, all previous studies have focused on symptomatic women, and there are no studies evaluating readmissions in women participating in screening programs. Given the increasing number of women participating in breast cancer screening programs, and therefore with cancers diagnosed at earlier stages [21], information on treatment complications, readmissions and health services’ use in this subset of women may be useful to provide more accurate information to women and to better forecast the use of health services.

The aim of this study was to explore risk factors associated with early and late readmissions in women diagnosed with breast cancer participating in screening programs, taking into account women’s and tumor characteristics, detection mode, the treatments received, and treatment-related complications.

## Materials and methods

### Setting and study population

We conducted a retrospective cohort study in a sample of 1086 women with breast cancer who underwent breast cancer screening in 4 screening programs in Spain (Parc de Salut Mar, Girona, Sabadell and Canary Islands), who were diagnosed with breast cancer between 2000 and 2009, and who were followed-up until June 2014. This cohort encompasses information on tumors diagnosed by routine screening mammograms and during the interval between 2 mammograms (CAMISS Cohort; ClinicalTrials.gov NCT03165006).

Following the recommendations of the European Guidelines for quality assurance in breast cancer screening and diagnosis [22, 23], the Spanish breast cancer screening program offers free biennial mammograms to women aged 50–69 years. Two-view mammography is performed and a double reading is made using the BI-RADS (Breast Imaging Reporting and Data System) classification or equivalent [24]. Once a tumor has been confirmed histologically, women enter the hospital circuit for treatment and follow-up and are no longer invited to participate in screening.

Data were collected through a protocol approved by the clinical research ethics committee of Parc de Salut Mar (Barcelona), and the rest of participant institutions. No informed consent was required.

### Readmission

For the purpose of our study, readmission was defined as a hospital admission related to the disease and/or complications of treatment that occurred at the same hospital where the patient was diagnosed and treated for breast cancer.

Readmission time (in days) was calculated from the date of the surgical treatment and the date of the first readmission. For those women not undergoing surgery (*n* = 6), the date of the first treatment (which was chemotherapy in all patients) was used to calculate the readmission date. We classified readmissions as: early readmissions (those occurring in the first 30 days after first treatment), late readmissions (between 30 days and 1 year after first treatment), and long-term readmissions (> 1 year after first treatment).

We also collected the reason for readmission associated with the disease, categorized as: tumor re-excision, scar dehiscence, drainage of an abscess, disease progression, complications due to adjuvant treatment, and others (including scar dislocation and abscess drainage).

**Fig. 1:**
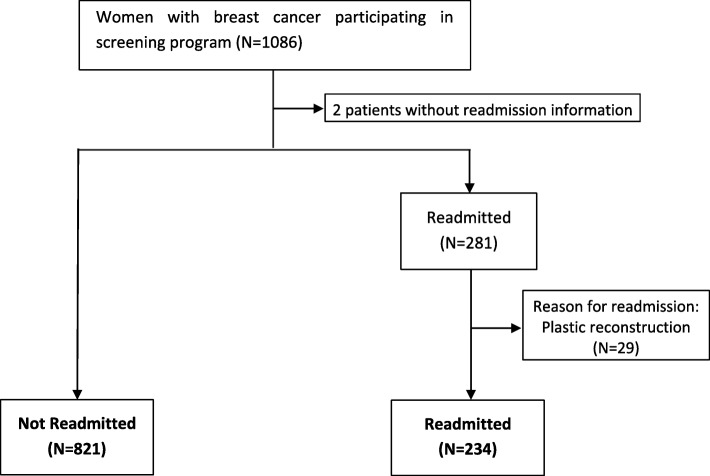
Flowchart of the study

We excluded women with no available information (*n* = 2) on readmissions. We also excluded women readmitted for plastic reconstruction (*n* = 29), since reconstructions were performed immediately in some hospitals and were deferred in others. Finally, we included 234 women with readmissions and 821 without readmissions (Fig. 1).

### Study variables

Women’s characteristics were obtained from the screening program databases, hospital-based tumor registries, population-based cancer registries, hospital administrative databases, and clinical records review. Age at diagnosis was calculated from the date of birth and the date of cancer diagnosis. The presence of comorbidities at diagnosis was obtained from the clinical records review. To measure the burden of disease, the Charlson Comorbidity Index (CCI) was used [25], collapsed into 2 categories: CCI = 0; CCI ≥1.

Information on the breast cancer detection mode was obtained from the databases of the screening programs. We differentiated between breast cancers detected by routine screening mammograms (i.e., screen-detected cancers) and cancers detected between 2 screening mammograms (i.e., interval cancers).

Tumor-related information was obtained from the population-based cancer registries, the hospital-based cancer registry, and the clinical records review. Information was collected on invasiveness (ductal carcinoma in situ, invasive cancer); focality (unifocal and multifocal and/or multicentric); tumor-node-metastasis (TNM) stage (stage 0-IV); tumor grade (well differentiated, moderately differentiated, and poorly differentiated/undifferentiated); and tumor phenotype. Tumors were classified into 4 phenotypes according to the expression of hormone receptors [estrogen receptor (ER) and progesterone receptor (RP)] and epidermal growth receptor 2 (Her2): luminal A (ER + / Her2- or PR + / Her2-); luminal B (ER + / Her2 + or PR + / Her2 +); Her2 (ER- / PR- / Her2 +); and triple-negative (ER-, PR-, Her2-) [26]. Ki67 expression was not taken into account to define phenotypes.

Treatment-related information was obtained from the clinical records review. The surgical approach was categorized as: 1) conservative surgery without lymphadenectomy; 2) conservative surgery with lymphadenectomy; 3) radical surgery with or without lymphadenectomy; and 4) no surgery and/or adjuvant treatment. Adjuvant treatment combinations were categorized as: 1) chemotherapy, radiotherapy, and hormonal therapy; 2) radiotherapy and hormone therapy; 3) radiotherapy and chemotherapy; 3) chemotherapy, radiotherapy, and hormone therapy; and 4) other treatments. Anti-Her 2 therapy could not be included in our analysis. Information on neoadjuvant and adjuvant treatments was also collected.

### Follow-up information

Data on complications associated with treatment, recurrences, and vital status at the end of the follow-up were obtained through the clinical records review. We categorized treatment-related complications as follows: general complications (including pain and anxiety), surgical complications (including lymphedema, adhesions, skin infection, and soft tissue necrosis), and medical complications (including treatment toxicity, endometrial alterations, hypothyroidism, mycosis, vascular insufficiency, asthenia, palpitations, mastitis, and depression). Recurrences included local, regional, and distant metastasis. Vital status at the end of follow-up was classified as alive or dead.

### Statistical analysis

Univariate and bivariate descriptive analyzes were performed comparing women’s and tumoral characteristics, complications, and the treatments received between women with and without readmission. We differentiated between early, late and long-term readmissions throughout the study. Statistical significance was estimated using the chi-squared test. For those variables showing statistically significant differences, two-sided equality tests for column proportions were calculated to assess which categories were statistically different.

To evaluate factors associated with early, late and long-term readmissions, we fitted 3 logistic regression models adjusted by age, Charlson Index, detection mode, TNM stage, focality, tumor grade, tumor phenotype, surgical and adjuvant treatment, general complications, surgical complications, medical complications, and screening program. Finally, additional analyses were performed, excluding complications from the final model.

Data management and statistical analyses were performed using the IBM SPSS Statistics program, version 25. Statistical significance was set at *p* < 0.05.

## Results

We included 1055 women diagnosed with breast cancer, of whom 76 had an early readmission (7.2%), 87 had a late readmission (8.2%), and 71 had long-term readmissions (6.7%) (Table 1).

**Table 1:** Women’s and tumoral characteristics according to readmission

	Readmitted, n (%) (*n* = 234)			Not readmitted, n (%) (*n* = 821)	p-value^1^
	Early readmission	Late readmission	Long-term readmission		
Total	76 (7.2)	87 (8.2)	71 (6.7)	821 (77.8)	
Age (years)					
50–54	24 (31.6)	22 (25.3)	32 (45.1)^a^	224 (27.3)^a^	0.013
55–59	25 (32.9)	27 (31.0)	16 (22.5)	212 (25.8)	
60–64	21 (27.6)	19 (21.8)	17 (23.9)	225 (27.4)	
65–69	6 (7.9)	19 (21.8)	6 (8.5)	160 (19.5)	
Missing	0	0	0	0	
Charlson index					
0	61 (80.3)	61 (70.1)	47 (66.2)	603 (73.4)	0.245
1	15 (19.7)	26 (29.9)	24 (33.8)	218 (26.6)	
Missing	0	0	0	0	
Detection mode					
Screen-detected	50 (65.8)	62 (71.3)	42 (59.2)	568 (69.2)	0.306
Interval Cancer	26 (34.2)	25 (28.7)	29 (40.8)	253 (30.8)	
Missing	0	0	0	0	
Tumor-node-metastasis stage					
In situ	6 (8.1)	8 (9.6)	5 (7.4)	84 (10.4)	0.069
Stage I	36 (48.6)^a^	38 (45.8)	18 (26.5)^a,b^	368 (45.5)^b^	
Stage II	25 (33.8)	26 (31.3)	28 (41.2)	249 (30.8)	
Stage III/IV	7 (9.5)	11 (13.3)	17 (25.0)^a^	108 (13.3)^a^	
Missing	2	4	3	9	
Focality					
Unifocal	43 (70.5)^a^	54 (75.0)	49 (81.7)	594 (85.3)^a^	0.005
Multifocal and/or multicentric	18 (29.5)^a^	18 (25.0)	11 (18.3)	102 (14.7)^a^	
Missing	15	15	11	125	
Tumor grade					
Well differentiated	13 (21.0)	18 (27.3)^a^	4 (7.0)^a,b^	193 (29.2)^b^	0.001
Moderately differentiated	33 (53.2)	34 (51.5)	27 (47.4)	248 (37.6)	
Poorly differentiated/ undifferentiated	16 (25.8)	14 (21.2)^a^	26 (45.6)^a^	219 (33.2)	
Not applicable	7	4	1	49	
Missing	7	17	13	112	
Tumor phenotype					
Luminal A	29 (58.0)	35 (64.8)	19 (41.3)	324 (51.8)	0.017
Luminal B	13 (26.0)	9 (16.7)	10 (21.7)	181 (28.9)	
HER2	3 (6.0)	3 (5.6)	11 (23.9)^a^	51 (8.1)^a^	
Triple-negative	5 (10.0)	7 (13.0)	6 (13.0)	70 (11.2)	
Missing	26	37	25	195	

^1^ Chi-square test

^a,b^ Values in the same row and subtable sharing the same subscript were significantly different (*p* < .05) in the two-sided equality test for column proportions

Women’s and tumor characteristics were compared between women with early, late, and long-term readmissions and those of women with no readmissions (Table 1). Age tended to be younger (< 60 years) in women with early readmissions than in those with no readmissions (64.5% vs 53.1%; *p* = 0.013). The percentages of multifocal and moderately differentiated tumors was higher in women with early readmissions than in those with no readmissions (*p* = 0.05). The percentage of luminal A tumors was higher in women with early and late readmissions than in those women with no readmissions (58.0%, 64.8 vs 51.8%, respectively), while the percentage of Her2 tumors was higher in women with long-term readmissions than in those with no readmissions (23.9 vs 8.1, respectively; *p* = 0.017). Although not statistically significant, women with readmissions tended to present with cancer at advanced TNM stages, and specifically with higher percentages of lymph node involvement (data not shown). No significant differences were found according to comorbidities at diagnosis or detection mode.

The treatments received according to type of readmission are summarized in Table 2. Women with long-term readmissions showed lower percentages of conservative surgery without lymphadenectomy than other study groups (*p* < 0.001), and higher percentages of radical surgery (*p* < 0.001). Women with long-term readmissions were more likely to have received neoadjuvant treatments (*p* < 0.001), whereas no statistically significant differences were found according to the adjuvant treatments received. However, chemotherapy, radiotherapy and hormone therapy were more common in women with early and late readmissions (39.5 and 32.2%; respectively), while radiotherapy and hormone therapy were more common in women not requiring readmission (35.4%).

**Table 2:** Treatments provided according to readmission

	Readmitted, n (%)			Not readmitted, n (%) (*n* = 821)	p-value^1^
	Early readmission (*n* = 76)	Late readmission (*n* = 87)	Long-term readmission (*n* = 71)		
Neoadjuvant therapy					
No	72 (94.7)^a^	80 (92.0)^b^	52 (73.2)^a,b,c^	731 (89.1)^c^	< 0.001
Yes	4 (5.3)^a^	7 (8.0)^b^	19 (26.8) ^a,b,c^	89 (10.9)^c^	
Missing	0	0	0	1	
Surgical treatment					
Conservative^2^ surgery without lymphadenectomy	30 (40.5)^a^	30 (34.9)^b^	10 (14.5) ^a,b,c^	267 (32.8)^c^	0.001
Conservative^2^ surgery with lymphadenectomy	27 (36.5)	40 (46.5)	31 (44.9)	386 (47.4)	
Radical^3^ surgery with or without lymphadenectomy	17 (23.0)	13 (15.1)^a^	25 (36.2) ^a,b^	149 (18.3)^b^	
No surgery and/or adjuvant treatment	0 (0.0)	3 (3.5)	3 (4.3)	12 (1.5)	
Missing	2	1	2	7	
Adjuvant treatment post-surgery					
Chemotherapy, radiotherapy and hormone therapy with/without anti-Her2 therapy	30 (39.5)	28 (32.2)	20 (29.0)	229 (28.1)	0.159
Radiotherapy and hormone therapy with/without anti-Her2 therapy	14 (18.4)	27 (31.0)	22 (31.9)	288 (35.4)	
Radiotherapy and chemotherapy with/without anti-Her2 therapy	9 (11.8)	5 (5.7)	9 (13.0)	71 (8.7)	
Other treatments	23 (30.3)	27 (31.0)	18 (26.1)	226 (27.8)	
Missing	0	0	2	7	

^1^ Chi-square test

^2^ Included: quadrantectomy, tumorectomy and segmentectomy

^3^ Included: simple mastectomy, radical mastectomy, and modified radical mastectomy

^a,b,c^ Values in the same row and subtable sharing the same subscript were significantly different (*p* < .05) in the two-sided equality test for column proportions

The percentages of patient complications associated with treatment, recurrences and vital status according to type of readmission are shown in Table 3. Women with readmissions showed higher percentages of general and medical complications than those without readmissions. The most common medical complications associated with late and long-term readmission were treatment toxicity (*N* = 20 and *N* = 11, respectively) and fatigue (*N* = 11 and *N* = 16, respectively). In the case of early readmission was fatigue (*N* = 10).

**Table 3:** Patient complications associated with treatment, recurrence and vital status according to readmission

	Readmitted, n (%) (*n* = 234)			Not readmitted, n (%) (*n* = 821)	p-value^1^
	Early readmission (*n* = 76)	Late readmission (*n* = 87)	Long-term readmission (*n* = 71)		
Complications associated with treatment^2^					
Any complication^**3**^	34 (44.7)^a^	44 (50.6)^b^	38 (53.5)^c^	182 (22.2)^a,b,c^	< 0.001
General complications^4^	20 (26.3)^a^	21 (24.1)^b^	22 (31.0)^c^	105 (12.8)^a,b,c^	< 0.001
Surgical complications^5^	18 (23.7)	14 (16.1)	13 (18.3)	110 (13.4)	0.076
Medical complications^6^	22 (28.9)^a^	29 (33.3)^b^	26 (36.6)^c^	80 (9.7)^a,b,c^	< 0.001
Recurrence					
No recurrence	66 (86.8)^a^	71 (81.6)^b^	16 (22.5)^c^	743 (90.5)^a,b,c^	< 0.001
Recurrence	10 (13.2)^a^	16 (18.4)^b^	55 (77.5)^c^	78 (9.5)^a,b,c^	
Vital status					
Alive	66 (86.8)^a^	75 (86.2)^b^	30 (42.3)^c^	728 (88.7)^a,b,c^	< 0.001
Dead	10 (13.2)^a^	12 (13.8)^b^	41 (57.7)^c^	93 (11.3)^a,b,c^	

^1^ Chi-square test

^2^ The categories are not exclusive as the same patient could have more than one complication

^3^ Number of women with at least one complication

^4^ Included: pain and anxiety

^5^ Included: lymphedema, adhesions, skin infection and soft tissue necrosis

^6^ Included: treatment toxicity, endometrial alterations, hypothyroidism, mycosis, vascular insufficiency, asthenia, palpitations, mastitis, and depression

^a,b,c^ Values of the same row and subtable that share the same subscript are significantly different in *p* < .05 in the two-sided equality test for column proportions

Women with early readmissions showed a higher proportions of surgical complications than other study groups, although this difference was not statistically significant (*p* = 0.076). In addition, recurrence and death were significantly more frequent in patients with early, late, and long-term readmissions than in those without readmissions.

The reasons for readmission associated with disease across readmission subgroups are shown in Table 4. Over 80% of early readmissions were due to tumor re-excision and mastectomy/lymphadenectomy, whereas most long-term readmissions were related to disease progress (78.9%).

**Table 4:** Reason of readmission associated with the disease, according to time until readmission

	Early readmission, n (%) (*N* = 76)	Late readmission, n (%) (*N* = 87)	Long-term readmission, n (%) (*N* = 71)	p-value^1^
Tumor re-excision	27 (35.5)^a^	26 (29.9)^b^	4 (5.6)^a,b^	< 0.001
Mastectomy and/or lymphadenectomy	36 (47.4)^a^	34 (39.1)^b^	3 (4.2)^a,b^	
Due to medical treatment	5 (6.6)	15 (17.2)	4 (5.6)	
Due to disease progression	3 (3.9)^a^	8 (9.2)^b^	56 (78.9)^a,b^	
Others (including scar dislocation, drain abscess, bleeding)	5 (6.6)	4 (4.6)	4 (5.6)	

Plastic surgery reconstructions were excluded because they differ between hospitals, some reconstruct at the time until the surgical intervention

^1^ Chi-square test

^a,b^ Values in the same row and subtable sharing the same subscript were significantly different (*p* < .05) in the two-sided equality test for column proportions

Multivariate analyses to assess risk factors for early, late and long-term readmissions, and adjustment by women’s and tumor characteristics, treatment received, complications, and screening program, are shown in Table 5. Conservative surgery without lymphadenectomy increased the risk of early readmission (aOR = 2.91; 95%CI = 1.13–7.52) compared with conservative surgery with lymphadenectomy. However, there was no association in women who underwent radical surgery. Surgical complications were associated with an increased risk of early readmission (aOR = 3.62; 95%CI = 1.27–10.29), but no association was found with women’s and tumor characteristics.

**Table 5:** Bivariate and multivariate logistic regression analysis of factors associated with early, late and long-term readmissions

	Early readmission Odds Ratio (95% CI)		Late readmission Odds Ratio (95% CI)		Long-term readmission Odds Ratio (95% CI)	
	Unadjusted	Adjusted^1^	Unadjusted	Adjusted^1^	Unadjusted	Adjusted^1^
Detection mode						
Screen-detected	Ref.	Ref.	Ref.	Ref.	Ref.	Ref.
Interval cancer	1.14 (0.70–1.86)	0.73 (0.32–1.66)	0.86 (0.53–1.40)	0.60 (0.23–1.53)	1.54 (0.94–2.53)	0.65 (0.22–1.88)
Tumor-node-metastasis stage						
In situ	0.73 (0.30–1.78)	1.44 (0.14–15.15)	0.94 (0.42–2.07)	2.55 (0.24–27.53)	1.25 (0.45–3.46)	5.21 (0.31–88.88)
Stage I	Ref.	Ref.	Ref.	Ref.	Ref.	Ref.
Stage II	0.97 (0.57–1.65)	0.65 (0.26–1.61)	0.96 (0.57–1.61)	1.93 (0.68–5.46)	2.29 (1.25–4.22)	0.93 (0.30–2.92)
Stage III/IV	0.61 (0.26–1.39)	0.27 (0.06–1.30)	0.93 (0.46–1.86)	2.71 (0.55–13.42)	3.31 (1.66–6.62)	1.64 (0.33–8.10)
Surgical treatment						
Conservative^2^ surgery without lymphadenectomy	1.65 (0.96–2.84)	2.91 (1.13–7.52)	1.09 (0.66–1.78)	1.26 (0.45–3.53)	0.45 (0.22–0.92)	0.46 (0.11–1.87)
Conservative^2^ surgery with lymphadenectomy	Ref.	Ref.	Ref.	Ref.	Ref.	Ref.
Radical^3^ surgery with or without lymphadenectomy	1.54 (0.82–2.89)	2.54 (0.84–7.71)	0.76 (0.40–1.45)	0.46 (0.12–1.76)	2.04 (1.17–3.55)	1.57 (0.46–5.30)
Surgical complications^4^						
No	Ref.	Ref.	Ref.	Ref.	Ref.	Ref.
Yes	1.91 (1.09–3.34)	3.62 (1.27–10.29)	1.13 (0.62–2.05)	1.23 (0.36–4.16)	1.33 (0.71–2.49)	0.59 (0.18–1.97)
Medical complications^5^						
No	Ref.	Ref.	Ref.	Ref.	Ref.	Ref.
Yes	2.55 (1.50–4.32)	2.69 (0.84–8.60)	3.28 (2.02–5.32)	8.72 (2.83–26.86)	3.76 (2.24–6.31)	4.79 (1.41–16.31)

^1^ Model adjusted by age, Charlson Index, detection mode, tumor-node metastasis stage, focality, tumor grade, tumor phenotype, surgical and adjuvant treatment, general complications, surgical and medical complications, and screening program

^2^ Included: quadrantectomy, tumorectomy, and segmentectomy

^3^ Included: simple mastectomy, radical mastectomy, and modified radical mastectomy

^4^ Included: lymphedema, adhesions, skin infection, and soft tissue necrosis

^5^ Included: treatment toxicity, endometrial alterations, hypothyroidism, mycosis, vascular insufficiency, asthenia, palpitations, mastitis, and depression

Medical complications were associated with a significantly increased risk of late readmission (Table 5) (aOR = 8.72; 95%CI = 2.83–26.86), as well as with long-term readmission (aOR = 4.79; 95%CI = 1.41–16.31).

Additional analysis excluding complications showed non-significantly different results to those presented in our main analysis. In particular, there were no differences in the association between the surgical approach and the risk of early, late and long-term readmissions (Additional file 1: Table A).

## Discussion

We aimed to identify the risk factors associated with early, late and long-term readmissions in women diagnosed with breast cancer participating in screening programs. Our findings suggest that women who experienced surgical or medical complications after breast cancer treatment had an increased risk of readmission, taking into account the detection mode and the treatments received. Specifically, women with surgical complications had an increased risk of early readmissions, while those with medical complications had an increased likelihood of late and long-term readmissions.

In our study, descriptive data revealed that early readmission occurred in 7.2% of women. Other studies calculating the percentages of early readmission after breast cancer surgery have found lower percentages of around 5.5% [15, 17]. The reason for this variability in the percentage of readmissions may be that these studies included women with broader age ranges and readmissions were analyzed after specific surgical procedures. A study exploring the variation in 30-day readmissions after a major surgical procedure, concluded that despite considerable variation in early readmission across surgical subspecialties, variation in early readmission was attributable to non-modifiable patient-level factors [16].

Although there is no single definition of readmission [27], most studies on readmissions among women with breast cancer have analyzed factors related to those occurring 30 days [15–17] or 1 year after surgical treatment [28]. Our study is consistent with prior series in showing an association between surgical complications (mainly wound complications and surgical-site infections) and early readmissions [14, 16]. An analysis of risk factors related to readmission after immediate breast reconstruction surgery found that patients with surgical complications had a 4-fold increased risk of early readmission [15]. Another study found that most early readmissions after mastectomy were related to postoperative complications, rather than exacerbations of comorbidities [17]. Other studies have shown that additional risk factors for early readmission were length of stay, payer type, physician volume, and active smoking [17, 18].

In the current adjusted analysis, which included surgical and medical complications, the risk of early readmission was higher in women receiving conservative surgery without lymphadenectomy than in those receiving conservative surgery with lymphadenectomy. In addition, most early readmissions were due to tumor re-excision, mastectomy or lymphadenectomy, which were probably related to previous conservative surgery which lately required a more aggressive approach. Furthermore, when we excluded complications from the main analysis, the risk of early readmission was not associated with the surgical approach. Therefore, it seems that early readmission risk was not increased by the surgical approach but rather by the complications themselves.

Late and long-term readmissions were more common in women with medical complications than in the other study groups, and in the adjusted analyses medical complications became a risk factor for late and long-term readmission regardless of the treatments received. Most medical complications consisted of the adverse effects of chemotherapy, such as fever, pain, nausea, vomiting, diarrhea, myelosuppression (neutropenia and leukopenia), and anemia [29, 30]. Only one study has evaluated readmissions after neoadjuvant chemotherapy in patients with breast cancer and, in the adjusted analyses, found that neoadjuvant chemotherapy was not statistically significantly associated with early readmissions [19]. Another study examined the reasons for, and factors associated with, early readmission after curative chemotherapy for breast cancer and reported that the factors associated with early readmission included tumor size (patients with T2 were more likely to be admitted), receiving adjuvant therapy versus neoadjuvant therapy, and undergoing fewer chemotherapy cycles [30]. It may be advisable to provide more intensive follow-up to women with complications due to chemotherapy in order to avoid unplanned readmissions and improve the use of health services for this subset of women.

In contrast with other studies, we found no association between comorbidities and readmissions [15–17]. This lack of association may be due to some misinformation, given that data on comorbidities were manually obtained from the clinical records review. Regarding detection mode, we observed a non-significant trend for higher rates of early and long-term readmissions in women with interval cancers. However, this effect was attenuated in the adjusted models, indicating that both women whose cancer was detected symptomatically or asymptomatically had the same chance of readmission. These results contrast with evidence showing differences in tumor characteristics according to detection mode [31, 32]. To our knowledge, this is the first work assessing factors associated with readmissions among screened women. However, to better assess the effect of breast cancer screening on readmissions, it would be interesting to compare cohorts of screened vs unscreened women.

This study has some limitations. First, the number of events in some categories of the analysis was relatively small, hampering the identification of significant associations. Nevertheless, the number of cases included in the study ensured sufficient statistical power to meet the study objectives. Second, the manual and retrospective collection of some variables might have introduced an information bias, either due to some misinformation or to variability in the quality of the information in the clinical records in the distinct hospitals. However, the clinical records review was done by trained professionals, following a common protocol, and the final models were adjusted by different screening programs to control the variability produced by the inclusion of distinct programs. Third, other relevant variables such as obesity, smoking status, breast reconstruction approach, or readmission not related to breast cancer could not be explored because they were not collected in the CAMISS cohort. However, we did include a number of variables related to tumor characteristics, treatments and complications, allowing us to provide an overview of all disease approach. Fourth, Ki67 expression was not included in the definition of phenotypes, since this information was not available in patients diagnosed at the beginning of the study period. Last, because treatment for Her2 tumors was introduced in 2006, the effect of this treatment could not be examined throughout the analysis. However, women with Her2-positive tumors showed higher percentages of long-term readmission, which were probably associated with recurrences in women who could not benefit from anti-Her2 treatment.

## Conclusion

In conclusion, our results suggest that the presence of surgical and medical complications increases the risk of early and late readmissions, adjusted by detection mode and treatments received. To our knowledge, this is the first study that analyzes the factors associated with readmissions among women participating in screening. This information may be useful to improve the management of the disease, especially among women with complications due to breast cancer treatment and predict health services use. Providing more intensive surveillance in women with treatment complications may help reduce further readmissions associated with the disease.

## Supplementary information

**Additional file 1. Table A.** Multivariate logistic regression analysis of factors associated with early, late and long-term readmissions.

## Data Availability

The datasets analysed during the current study are not publicly available due to privacy regulations, but are available from the corresponding author on reasonable request.

## Abbreviations

aOR: Adjusted odds ratio
BI-RADS: Breast Imaging Reporting and Data System
CCI: Charlson Comorbidity Index
CI: Confidence intervals
ER: Estrogen receptor
Her2: Human epidermal growth factor receptor 2
OR: Odds ratio
PR: Progesterone receptor
TNM: Tumor-node-metastasis
