# Supplementary material for: Factors associated with readmissions in women participating in screening programs and treated for breast cancer: a retrospective cohort study
Source: BMC Health Serv Res. 2019 Dec 5;19:940. doi: 10.1186/s12913-019-4789-3 (PMC6896282; doi:10.1186/s12913-019-4789-3)
Supplement: Supplementary file 1 — Additional file 1. Table A. Multivariate logistic regression analysis of factors associated with early, late and long-term readmissions. [file 12913_2019_4789_MOESM1_ESM.doc]

**Supplementary material**

**Table A. Multivariate logistic regression analysis of factors associated with early, late and long-term readmissions**

|  | **Early readmission**  Odds Ratio (95% CI) | | **Late readmission**  Odds Ratio (95% CI) | | **Long-term readmission**  Odds Ratio (95% CI) | |
| --- | --- | --- | --- | --- | --- | --- |
|  | **Unadjusted** | **Adjusted^1^** | **Unadjusted** | **Adjusted^1^** | **Unadjusted** | **Adjusted^1^** |
| **Detection mode** | | | | | | |
| Screen-detected | Ref. | Ref. | Ref. | Ref. | Ref. | Ref. |
| Interval cancer | 1.14 (0.70-1.86) | 0.83 (0.38-1.82) | 0.86 (0.53-1.40) | 0.80 (0.34-1.93) | 1.54 (0.94-2.53) | 0.75 (0.28-2.05) |
| **TNM Stage** | | | | | | |
| In situ | 0.73 (0.30-1.78) | 1.21 (0.12-12.46) | 0.94 (0.42-2.07) | 2.79 (0.26-29.56) | 1.25 (0.45-3.46) | 5.10 (0.31-83.27) |
| Stage I | Ref. | Ref. | Ref. | Ref. | Ref. | Ref. |
| Stage II | 0.97 (0.57-1.65) | 0.70 (0.29-1.69) | 0.96 (0.57-1.61) | 1.68 (0.61-4.61) | 2.29 (1.25-4.22) | 0.94 (0.31-2.86) |
| Stage III/IV | 0.61 (0.26-1.39) | 0.31 (0.07-1.43) | 0.93 (0.46-1.86) | 2.44 (0.54-11.02) | 3.31 (1.66-6.62) | 1.81 (0.40-8.15) |
| **Surgical Treatment** | | | | | | |
| Conservative^2^ surgery without lymphadenectomy | 1.65 (0.96-2.84) | 2.23 (0.89-5.54) | 1.09 (0.66-1.78) | 1.00 (0.38-2.63) | 0.45 (0.22-0.92) | 0.47 (0.12-1.86) |
| Conservative^2^ surgery with lymphadenectomy | Ref. | Ref. | Ref. | Ref. | Ref. | Ref. |
| Radical^3^ surgery with or without lymphadenectomy | 1.54 (0.82-2.89) | 2.25 (0.89-5.54) | 0.76 (0.40-1.45) | 0.41 (0.11-1.48) | 2.04 (1.17-3.55) | 1.52 (0.49-4.77) |

^1^ Model adjusted by age, Charlson Index, detection mode, TNM stage, focality, tumor grade, tumor phenotype, surgical and adjuvant treatment, and screening program.

^2^ Included: quadrantectomy, tumorectomy and segmentectomy.

^3^ Included: simple mastectomy, radical mastectomy and modified radical mastectomy.
